# Ameliorative Effects of *Zingiber officinale* Rosc on Antibiotic-Associated Diarrhea and Improvement in Intestinal Function

**DOI:** 10.3390/molecules29030732

**Published:** 2024-02-05

**Authors:** Sung Jin Kim, Myoung-Sook Shin, You-Kyung Choi

**Affiliations:** College of Korean Medicine, Gachon University, Seongnam 13120, Republic of Korea; sungjinkim001@gmail.com (S.J.K.);

**Keywords:** antibiotic-associated diarrhea, intestinal barrier, short-chain fatty acid, *Zingiber officinale* Roscoe

## Abstract

The global increase in antibiotic consumption is related to increased adverse effects, such as antibiotic-associated diarrhea (AAD). This study investigated the chemical properties of *Zingiber officinale* Rosc (ZO) extract and its ameliorative effects using a lincomycin-induced AAD mouse model. Intestinal tissues were evaluated for the expression of lysozyme, claudin-1, and α-defensin-1, which are associated with intestinal homeostasis. The cecum was analyzed to assess the concentration of short-chain fatty acids (SCFAs). The chemical properties analysis of ZO extracts revealed the levels of total neutral sugars, acidic sugars, proteins, and polyphenols to be 86.4%, 8.8%, 4.0%, and 0.8%, respectively. Furthermore, the monosaccharide composition of ZO was determined to include glucose (97.3%) and galactose (2.7%). ZO extract administration ameliorated the impact of AAD and associated weight loss, and water intake also returned to normal. Moreover, treatment with ZO extract restored the expression levels of lysozyme, α-defensin-1, and claudin-1 to normal levels. The decreased SCFA levels due to induced AAD showed a return to normal levels. The results indicate that ZO extract improved AAD, strengthened the intestinal barrier, and normalized SCFA levels, showing that ZO extract possesses intestinal-function strengthening effects.

## 1. Introduction

At the beginning of the 20th century, infectious diseases such as smallpox, measles, and pneumonia were widely prevalent and considered a leading cause of death worldwide [1]. In such serious situations, the introduction of antibiotics significantly decreased the incidence of mortality owing to infectious diseases. However, in addition to such changes, side effects due to the overuse of antibiotics and antibiotic resistance became problematic [2]. A study by Klein et al. analyzed the trends for antibiotic consumption between 2000 and 2015 in 76 countries and reported that the total antibiotic consumption rate increased by 39% [3]. Several studies have reported that the long-term use of antibiotics resulted in various side effects, such as hypersensitivity, direct toxic effects on tissues, and antibiotic-associated diarrhea, which is caused by antibiotics that can kill good bacteria and cause new infections [4,5]. Currently used antibiotics can be classified into different groups depending on their mechanism of action, chemical structure, and antimicrobial spectrum [6]. Among these antibiotics, lincomycin causes a change in the normal flora and induces diarrhea due to *Clostridium difficile* overgrowth [7]. *C. difficile* is a Gram-positive, spore-forming, anaerobic bacterium, which normally resides in small amounts in the intestinal tract. With the use of antibiotics, the levels of this bacterial flora increase, leading to diarrhea or infection caused by inflammation of the intestinal mucosa [8]. Major complications of AAD are associated with functional impairment of the intestinal wall. The intestinal wall consists of the mucosa, epithelium, and lamina propria, which form protein complexes called tight junctions (TJs), and play a role in protecting the intestine from harmful substances from the external environment and pathogens [9]. TJs connect intestinal epithelial cells and are composed of proteins such as claudin-1 and occluding, depending on the location of epithelium and permeability [10]. However, AAD-induced intestinal barrier impairment increases epithelial permeability, resulting in leakage of the gut and inflammation [11]. In the intestinal mucosal layer, bacterial flora interact with Paneth cells to secrete immunomodulatory substances, such as defensin, lysozyme, C-type lectins, and immunoglobulin A (IgA) [12]. However, the reduction in bacterial flora levels due to the use of antibiotics also reduces the secretion of antimicrobial substances, resulting in impaired intestinal immune function [13]. Furthermore, most carbohydrates are absorbed in the small intestine. However, some carbohydrates are fermented by normal flora and transformed into short-chain fatty acids (SCFAs), such as acetic acid, propionic acid, butyric acid, etc. [9]. The decreased levels of normal flora that result from the use of antibiotics cause an excessive amount of non-digestible carbohydrates to remain in the intestines, allowing them to move to the colon and absorb water via osmosis, ultimately leading to osmotic diarrhea [10].

Recently, studies have demonstrated that natural and plant-derived polysaccharides can help improve diarrhea and intestinal immunity [14,15,16,17]. Moreover, we have recently reported that the oral administration of ZO extract activates immunocytes in the Peyer’s patch, an intestinal lymphoid tissue, to increase the production of granulocyte–macrophage colony-stimulating factor and IgA in mice [18]. Ginger (*Zingiber officinale* Rosc.) is a perennial herbaceous plant native to tropical or subtropical regions and belongs to the Zingiberaceae family. It refers to the rhizome, or root stem, of the ginger plant [19]. The major components of ginger include carbohydrates (50–70%), lipids (3–8%), terpenes, and phenolic compounds such as gingerol, shogaol, and paradols. Additionally, it contains amino acids, fiber, proteins, phytosterols, vitamins (e.g., nicotinic acid and vitamin A), and minerals [20,21,22]. The terpenes in ginger, such as zingiberene and γ-cardinene, contribute to its distinctive smell [22]. The main components representing the pharmacological activity of ginger are known as 6-gingerol and 6-shogaol. These compounds exhibit antioxidant and anti-inflammatory properties, with 6-gingerol specifically showing antioxidant activity equivalent to 95% of ascorbic acid [23,24]. Furthermore, 6-shogaol has been proven to have various physiological effects, including antibacterial action [25], anti-inflammatory effects [26], anti-obesity properties [27], and activation of innate immunity [28,29]. However, the ameliorative effects of ZO extract on AAD and its mechanism of action have not yet been investigated. Therefore, this study evaluated the ameliorative effects of ZO extract on lincomycin-induced diarrhea and analyzed the levels of intestinal proteins and SCFAs to investigate its effects on intestinal health.

## 2. Results and Discussion

### 2.1. Chemical Properties of ZO Extract

In a previous study, six major index ZO compounds (6-Gingerol, 8-Gingerol, 6-Shogaol, 10-Gingerol, 8-Shogaol, and 10-Shogaol) were analyzed, with concentrations of 6.64, 0.22, 0.28, 0.16, and 0.01 mg/g, respectively, and the limit of quantitation was identified [18]. Furthermore, these six index compounds did not affect the proliferation of Peyer’s patch cells, GM-CSF, and IgA secretion.

Plant cell walls contain polysaccharides known as pectins, composed of homogalacturonan (HG) structures [30]. According to previous reports, polysaccharides can induce immune system activation, enhance intestinal immune function, and modulate the intestinal microbiome [15,31,32]. Therefore, we analyzed the chemical properties and composition of ZO extracts. As described in Table 1, the concentrations of total neutral sugar, acidic sugar, protein, and polyphenol in ZO extract were determined to be 86.4 ± 29.3, 8.8 ± 4.6, 4.0 ± 0.5, and 0.8 ± 1.1%, respectively. Furthermore, to analyze the monosaccharide composition of ZO, we derivatized the ZO extracts into 3-methyl-1-phenyl-2-pyrazolone (PMP) and analyzed them using high-performance liquid chromatography with ultraviolet detection (UV-HPLC) (Figure S1). The results indicated a glucose content of 97.3 ± 0.6% and galactose content of 2.7 ± 0.9 mole%. Thus, the ZO extract is primarily composed of neutral sugars, specifically glucose and galactose, with glucose being the main monosaccharide in ZO extracts.

### 2.2. Ameliorating Effect of ZO Extract on AAD in a Lincomycin-Induced-Diarrhea Mouse Model

**Figure 1:**
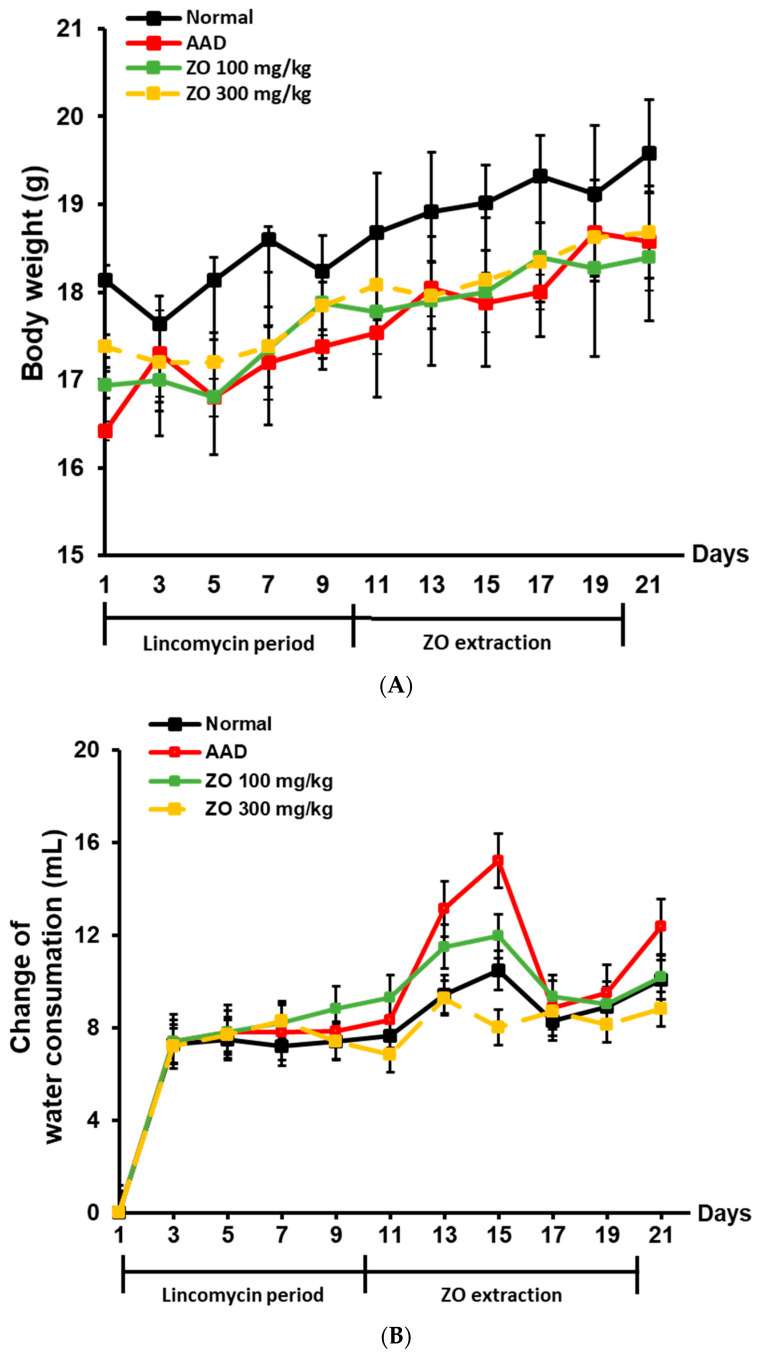

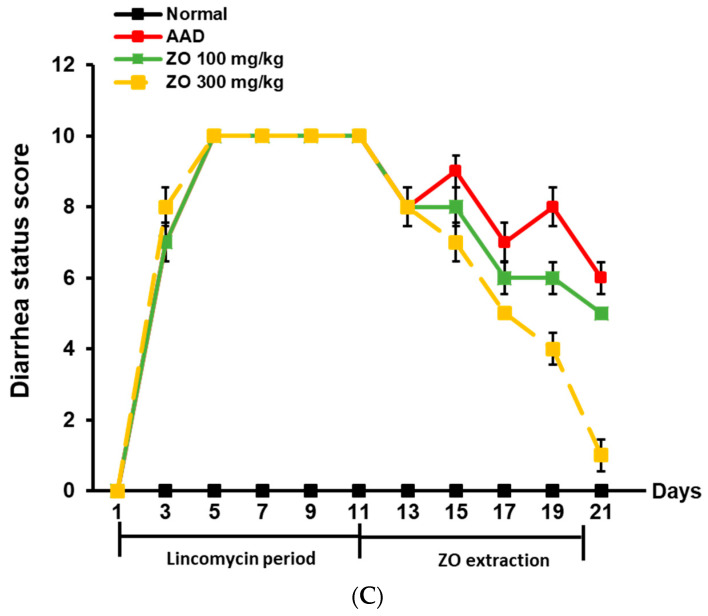
Effect of ZO extraction on changes in body weight, diarrhea status score, and water intake in the AAD model using BALB/c mice. AAD models were established by treatment with or without lincomycin via oral administration for ten days in mice. After diarrhea induction was completed, ZO extracts were orally administered at concentrations of 100 mg/kg or 300 mg/kg for ten days. Mouse body weight was measured every two days (**A**). Changes in the water intake of mice were recorded every other day (**B**). Diarrhea status scores of mice were observed once every 2 days (**C**).

To evaluate the ameliorative effects of ZO extract in an AAD mouse model, parameters including weight, diarrhea status score, and water intake were analyzed. During the treatment period using lincomycin, the rate of increase in weight was significantly lower in the group that received the antibiotic agent than in the normal group (Figure 1A). The group that received lincomycin showed an increase in water intake (Figure 1B). However, the rate of increase in weight was slightly higher in the ZO extract group than in the AAD group, confirming the likelihood of normalization. Furthermore, the increased water intake due to the use of lincomycin in the ZO extract group (100 and 300 mg/kg) was similar to the level of water intake of the normal group.

Figure 1C shows the total diarrhea status score evaluated according to the criteria listed in Table 2. The average diarrhea status score during the treatment period using lincomycin was 10 points, and the score dropped to 7 points when measured after treatment with lincomycin. Thereafter, the average diarrhea status scores in the ZO extract groups of 100 and 300 mg/kg were 6 and 1 point, respectively, indicating that ZO extract administration improved diarrhea. Based on these results, ZO extract ameliorated weight loss and diarrhea induced by lincomycin, and water intake.

### 2.3. Analysis of the Effects of ZO Extract on Changes in the Expression of Intestinal Lysozyme and Claudin-1 in the Lincomycin-Induced-Diarrhea Mouse Model

The impairment of mucosal barrier function plays an important role in the occurrence of intestinal leakage and inflammation [33]. Tight junction (TJ) proteins in the intestinal epithelium play a crucial role in maintaining intestinal homeostasis [34]. Lysozyme exerts an antimicrobial activity by damaging the cell surface and inducing cytolysis [35]. The enzyme is secreted by Paneth cells, which are epithelial cells in the intestinal mucosa [23]. Lysozyme also plays a role in suppressing intestinal bacterial proliferation [27,28]. Claudin-1 enhances the adhesion between intestinal epithelial cells and regulates the passage of substances in the intestine [29,36].

**Figure 2:**
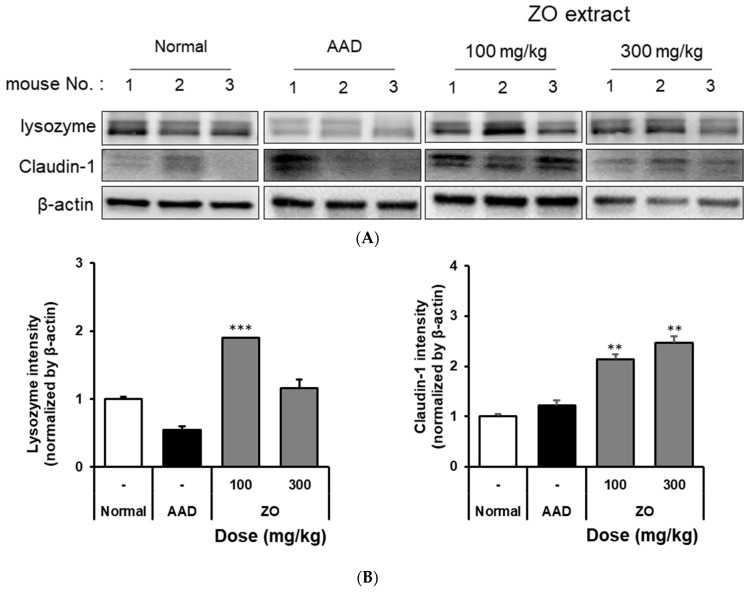
The effect of oral administration of ZO extract on lysozyme and claudin-1 expression in the AAD model. AAD mice were orally administered the ZO extract (100 mg/kg or 300 mg/kg) daily for ten days. The intestinal tissue was extracted using a radioimmunoprecipitation assay buffer. Lysozyme and claudin-1 protein expression were determined by immunoblotting. β-actin was used as an internal loading control (**A**). Lysozyme and claudin-1 expression were analyzed using Image J software (**B**). Data are presented as the mean ± standard deviation (SD) of triplicate experiments. ***, *p* < 0.0001 and **, *p* < 0.001 vs. the AAD group.

We evaluated whether ZO extract improves the damaged intestinal barrier induced by lincomycin administration. Figure 2 shows that the expression of lysozyme was significantly reduced in the AAD group compared to that in the normal group. In the ZO extract group, the expression of lysozyme significantly increased compared to that in the AAD group. Moreover, the expression of claudin-1 significantly decreased in the AAD group compared to that in the normal group. The decreased expression of claudin-1 was significantly improved in the ZO extract group. However, a dose-dependent phenotype with the ZO extract was not observed, possibly because the ZO extract was prepared from the crude polysaccharide fraction; thus, further purification of the ZO extract is required.

### 2.4. Analysis of the Effects of ZO Extract on the mRNA Expression of Intestinal Claudin-1, α-Defensin-1, and Lysozyme in the Lincomycin-Induced-Diarrhea Mouse Model

Claudin-1 is a TJ protein in the intestinal mucosa and plays a role in regulating its permeability [37]. Claudin-1 normally resides between epithelial cells and controls intercellular gaps, absorption, and the secretion of substances inside the small intestine [38]. Furthermore, α-defensin-1 is an antimicrobial peptide and is produced in the epithelial cells of the intestinal mucosa. This peptide plays a role in defending the small intestine from infection by interrupting bacterial growth and controlling the number of bacteria in the small intestine [39]. Lysozyme is produced in the intestinal mucosa and destroys the bacterial cell wall in the extracellular matrix [40].

**Figure 3:**
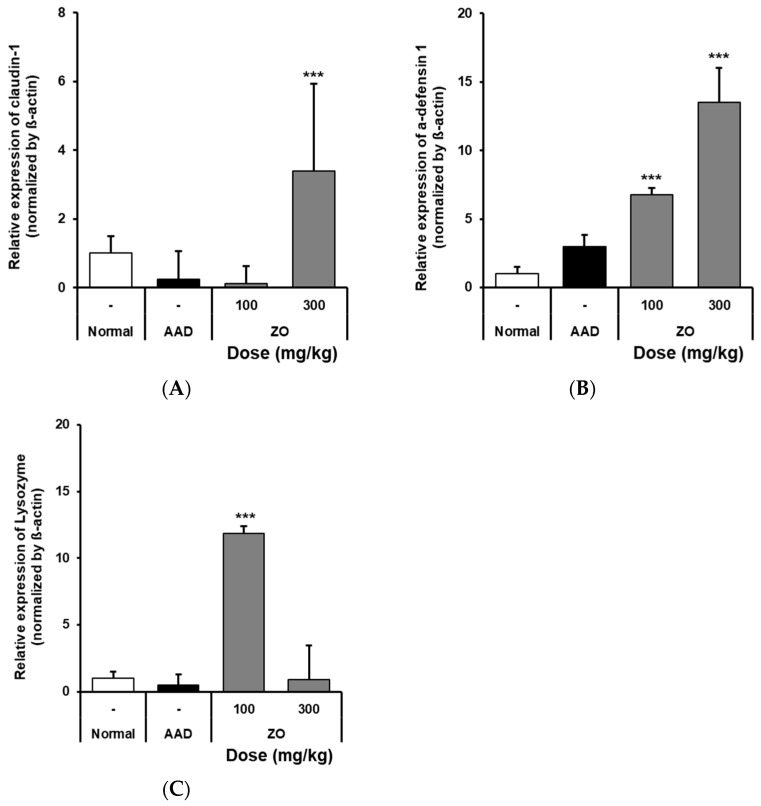
Effects of oral administration of ZO extract on lysozyme and claudin-1 mRNA expression in the AAD model. AAD mice were orally administered ZO extracts (100 mg/kg or 300 mg/kg) daily for 10 days. Intestinal RNA was extracted and claudin-1 (**A**), α-defensin1 (**B**), and lysozyme (**C**) mRNA expressions were determined by RT-qPCR. Data are presented as the mean ± standard deviation (SD) of triplicate experiments. ***, *p* < 0.0001 vs. the AAD group.

RT-qPCR was used to detect the mRNA expression of claudin-1, α-defensin-1, and lysozyme in the intestinal tissues of mice that received ZO extract in the lincomycin-induced-diarrhea model group. The results showed that the mRNA expression of claudin-1 in the AAD group was significantly reduced compared to that in the normal group. In contrast, in the group that received 300 mg/kg ZO extract, the mRNA expression of claudin-1 increased (Figure 3A). Further, the ZO extract group showed a dose-dependent increase in the expression of α-defensin-1 compared to that in the AAD group (Figure 3B). In the ZO extract group administered 100 mg/kg of ZO extract, the expression of lysozyme was significantly increased (Figure 3C). Based on these results, the use of ZO extract was shown to increase the mRNA expression of claudin-1, α-defensin-1, and lysozyme in the intestine of the lincomycin-induced-diarrhea model group.

### 2.5. Analysis of the Effects of ZO Extract on the Changes in the Levels of SCFAs in the Lincomycin-Induced-Diarrhea Model

Intestinal microorganisms use food ingested in the digestive tract to cause the fermentation of dietary fibers and the production of SCFAs, and they are affected by the physicochemical properties of dietary fibers [41]. SCFAs play a key role in maintaining the functions of epithelial cells and regulating the immune function [42]. Furthermore, SCFAs can alleviate diarrhea by absorption via colonic epithelial cells and stimulating the absorption of water and electrolytes [43]. Acetic, propionic, and butyric acids are well-known SCFAs. Acetic acid is a major SCFA that results due to lactic acid fermentation and is known to play an important role in inhibiting harmful bacteria in the intestine by reducing the intestinal pH, promoting immunocompetence, and regulating the intestinal environment [44] Butyric acid plays an important role in intestinal health by acting on colonocyte metabolism as a major SCFA, and is known to promote the proliferation and differentiation of intestinal epithelial cells and it helps prevent colon cancer [41,45]. Propionic acid is absorbed in the liver and inhibits cholesterol synthesis, and it strongly lowers blood cholesterol and lipid levels by regulating the expression of genes related to lipid synthesis enzymes [41]. 

**Figure 4:**
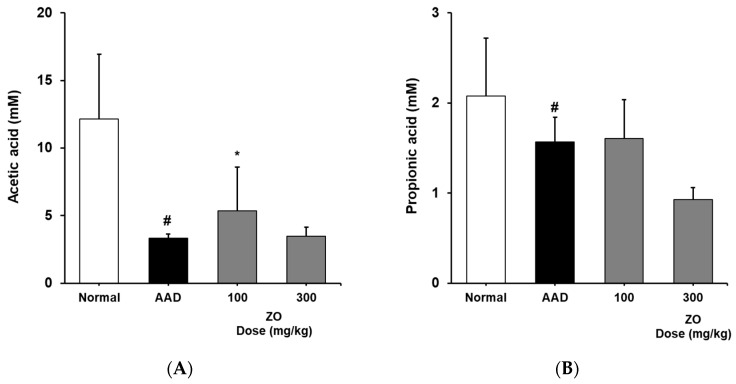

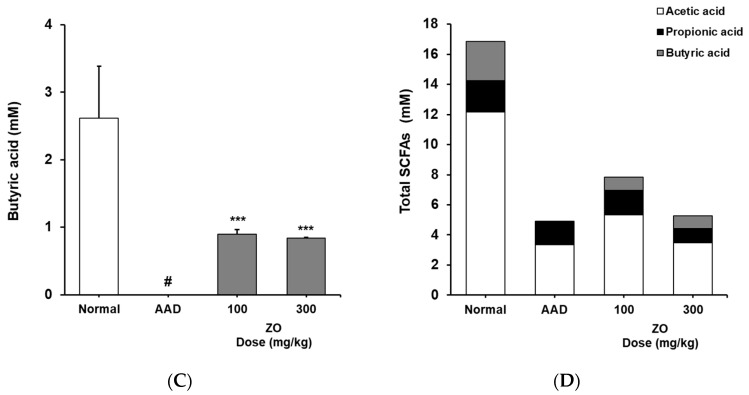
Effects of ZO extract on short-chain fatty acids in the cecum of lincomycin-induced AAD mice. AAD mice were orally administered the ZO extract (100 mg/kg or 300 mg/kg) daily for ten days. Acetic acid (**A**), butyric acid (**B**), propionic acid (**C**), and total SCFA content (**D**) in the mouse cecum were determined using flame ionization detector–gas chromatography. Data are presented as the mean±standard deviation (SD) of triplicate experiments. #, *p* < 0.05 vs. the normal group; ***, *p* < 0.05 vs. the AAD group and *, *p* < 0.01 vs. the AAD group.

We analyzed the effects of the ZO extract on SCFA levels in the appendix in the lincomycin-induced-diarrhea mouse model and found that the concentration of acetic acid in the AAD group decreased from 12.2 ± 4.8 to 3.3 ± 0.3 mM (Figure 4). In contrast, the concentrations of acetic acid in the groups that received 100 and 300 mg/kg of ZO extract were 5.3 ± 3.2 and 3.5 ± 0.7 mM, respectively. In the control group, the concentration of butyric acid was 2.6 ± 0.6 mM. In the AAD group, butyric acid was not detected. In the groups that received 100 and 300 mg/kg of ZO extract, the concentrations of butyric acid were 0.9 ± 0.1 and 0.8 ± 0.0 mM, respectively, indicating a recovery of butyric acid levels. The concentration of propionic acid in the AAD group decreased from 2.1 ± 0.6 to 1.6 ± 0.3 mM. No significant change in the concentration of propionic acid was found in the groups that received ZO extract compared to that in the AAD group (Figure 4). This result may be observed due to the effect of dietary fibers that contribute to the production of SCFAs and the composition of monosaccharides residing in crude polysaccharides, the major substance of ZO extract. Therefore, further studies on the composition of monosaccharides of ZO extract are required.

Based on these results, we infer that the use of ZO extract helps in recovering the reduced concentration of SCFAs in the lincomycin-induced-diarrhea mouse model and may contribute to the maintenance of intestinal homeostasis.

## 3. Materials and Methods

### 3.1. Preparation of ZO Extract

*Zingiber officinale* Roscoe (ZO) (30 g) was extracted with 300 mL of distilled water at 100 °C for 60 min. The extract was filtered through a non-woven fabric. Finally, 240 mL of supernatant was lyophilized using a freeze dryer (EYELA, Tokyo, Japan), and the yield was calculated by measuring the dried amount (Table 3).

### 3.2. Chemicals and Antibodies

To analyze the components of the extract, galactose (≥99%, Sigma-Aldrich, St. Louis, MO, USA) was used as a standard, and the neutral sugar content was measured using phenol–sulfuric acid [46]. Additionally, galacturonic acid (≥99%, Sigma-Aldrich, St. Louis, MO, USA) was used as a standard to measure acidic sugar content using *m*-hydroxybiphenyl [47]. The protein content was measured using the Bradford method with BSA (≥99%, Bio-Rad, Hercules, CA, USA) as the standard, and the phenolic compound content was quantitatively analyzed using gallic acid (≥99%, Sigma-Aldrich, St. Louis, MO, USA) as the standard via the Folin–Ciocalteu method [48,49]. The antibodies used included anti-lysozyme (Abcam, ab108508, Cambridge, UK), anti-claudin-1 (Abcam, ab180158), and anti-GAPDH (Cell Signaling Technology, #4967), and they were purchased from Cell Signaling Technology (Danvers, MA, USA). GAPDH levels were used as a loading control.

### 3.3. Monosaccharide Composition Analysis of ZO Extract

For sugar analysis, 2 M trifluoroacetic acid (TFA) from Sigma was added to ZO extract, and the reaction was incubated at 120 °C for 1 h, followed by hydrolysis and drying for 30 min. Next, 100 μL of 0.3 M NaOH and 120 μL of 0.5 M 3-methyl-1-phenyl-2-pyrazolone (PMP) in methanol were added, and the reaction was incubated at 70 °C for 1 h. The mixture was neutralized with 100 μL of 0.3 M HCl, thoroughly dried, and separated using a chloroform/H_2_O two-phase solvent system. The water layer was recovered, filtered, and analyzed by HPLC. The HPLC conditions are described in Table 4. The mole% of each constituent sugar was calculated by comparing the peak area and molecular weight of the sample relative to the internal standard. 

### 3.4. Animal and Experimental Design

**Figure 5:**
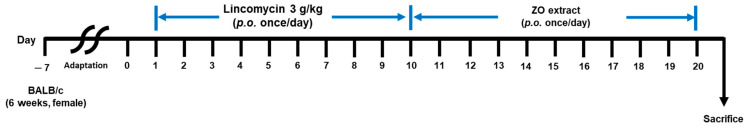
Oral administration schedule for ZO extraction in the lincomycin-induced AAD model. AAD models were induced by the administration of 3 g/kg lincomycin for ten days. CMC solutions were administered to the control groups. The ZO extraction group was orally administered 100 or 300 mg/kg lincomycin for ten days.

Animal experiments were conducted as per the guidelines of the Institutional Animal Care and Use Committee (IACUC) at Gachon University (Approval No: GU1-2022-IA0050-00). BALB/C mice (seven weeks, female) were purchased from OrientBio (Seoungnam, Korea). The experimental animals were exposed to a 12 h light–dark cycle and provided ad libitum access to food and water in an environment with a temperature of 22 ± 2 °C and humidity of 50–55%. After a 7-day adaptation period, the animals were assigned to the following groups: control (normal), antibiotic-induced-diarrhea (AAD), low-dose treatment (AAD + ZO 100 mg/kg), and high-dose treatment groups (AAD + ZO 300 mg/kg). Subsequently, the control group received oral administration of physiological saline, and the AAD group received oral administration of lincomycin (Dongkwang Pharm, Seoul, Korea) at a concentration of 3 g/kg once daily for ten days. After the completion of lincomycin administration, both the control and AAD groups received oral administration of sterilized 0.5 *w*/*v* % methyl cellulose 400 solution (CMC) (Wako, Tokyo, Japan). The ZO group was orally administered lincomycin for ten days and subsequently orally administered concentrations of 100 or 300 mg/kg for ten days (Figure 5).

### 3.5. Immunoblotting

Proteins from mouse small intestines were extracted in radioimmunoprecipitation assay buffer containing a phosphatase inhibitor cocktail (Sigma-Aldrich, St. Louis, MO, USA), 1 mM dithiothreitol (Wako, Tokyo, Japan), and a Complete™ Mini Protease Inhibitor Cocktail (Roche Diagnostics Corp., Indianapolis, IN, USA). The protein solution was centrifuged at 4 °C and 13,000 rpm for 20 min to separate the proteins from the intestinal tissues. After separating the proteins by SDS-PAGE, the proteins were transferred to a polyvinylidene difluoride (PVDF) membrane. The PVDF membrane was incubated with 5% skim milk overnight. Subsequently, specific antibodies were diluted in TBS with Tween 20 (0%) and applied to the membrane. The membrane was washed three times with TBS-T buffer. The membrane was incubated with a secondary antibody linked to horseradish peroxidase (HRP) at room temperature for 2 h. The protein signals were visualized with the Super Signal West Femto Substrate (Thermo Fisher, Emeryville, CA, USA) using the Fusion Solo Chemiluminescence System (Vilber Lourmat, Paris, France).

### 3.6. RT-qPCR

Mouse small intestines tissues were extracted using BioMasher (TaKaRa, Shiga, Japan) and filtrated using a QIAshredder (Qiagen, Hilden, Germany). Total RNA from the intestinal tissues was isolated and purified using the RNeasy Mini Kit (Qiagen, Hilden, Germany), followed by cDNA synthesis using the RevertAid First-Strand cDNA synthesis kit (Fermentas, MA, USA). The qRT-PCR quantification was performed using Mm02524428_g1(α-defensin-1), Mm01228299_m1 (lysozyme), and Mm01342184_m1 (claudin1) TaqMan primer sets (Applied Biosystems, Foster City, CA, USA). The amplification conditions were determined using the Quant 3 PCR system (Applied Biosystems).

### 3.7. Determination of Short-Chain Fatty Acids

To evaluate the SCFA content in the mouse cecum, we ground the tissue using the BioMasher (TaKaRa, Shiga, Japan) in an 80% methanol solution. The ground solution was then centrifuged at 13,000 rpm for 10 min at 4 °C, and the supernatant was filtered using ADVANTEC’s 0.45 μm syringe filter. Subsequently, analysis was performed using a flame ionization detector (HP-5890/5971, Hewlett Packard, Palo Alto, CA, USA) and GC column (DB-FFAP 123-3253, Agilent Technologies, Inc., Santa Clara, CA, USA, 50 mm × 0.32 mm × 0.50 μm). The SCFA levels were quantified using acetic acid, propionic acid, and butyric acid as standards. The content of SCFAs in the cecum was determined using a calibration curve based on the respective standards. Acetic acid, propionic acid, and butyric acid were purchased from Sigma-Aldrich (St. Louis, MO, USA).

### 3.8. Statistical Analysis

The results from the experiments were analyzed for significance using GraphPad Prism 5.02. The data are presented as the mean and standard deviation (SD) of triplicate experiments for all measured parameters. Significant differences between samples were determined using one-way ANOVA followed by Tukey’s multiple comparison analyses.

## 4. Conclusions

Currently, treatments such as the antibiotics metronidazole and vancomycin, and the anti-diarrheal agent loperamide, are used as therapeutic agents for antibiotic-associated diarrhea [50]. These treatments are known to have side effects such as allergic reactions, decreased appetite, development of resistant bacterial strains, and intestinal microbial imbalance [51,52,53,54]. Natural products are known to be relatively safe and have few side effects, and research is being conducted on the development of medicines using natural products. Currently utilized herbal medicines such as *Glycyrrhiza glabra*, *Allium sativum*, *Poria cocos*, and *Atractylodes* reportedly induce effects including alleviation of intestinal inflammation, antimicrobial effects, anti-diarrheal properties, and enhancement of digestive function [55,56,57,58].

This study analyzed the effects of ZO extract on AAD induced by lincomycin. Administration of a hot water extract of ZO was shown to improve diarrhea status, weight loss, and water intake in the AAD mouse model. Moreover, the expressions of lysozyme and α-defensin-1, the antimicrobial peptides of the small intestine, which were reduced due to AAD, were increased. An increase in the expression of claudin-1, the TJ protein, was also observed. Furthermore, the use of ZO extract improved the concentrations of intestinal SCFAs such as acetic and butyric acid, which were reduced due to AAD. 

The majority of polysaccharides generated through natural hot water extraction of ZO are pectins [59]. These polysaccharides primarily consist of homogalacturonan with a structure incorporating bound rhamnogalacturonan [60]. The pharmacological activity of pectin is largely attributed to subtle structural differences in rhamnogalacturonan. In this study, we conducted chemical and monosaccharide composition analyses of ZO extracts. Further research is warranted to elucidate the active components of ZO and undertake structural analyses of ZO polysaccharides through additional purification processes. To summarize, the hot water extract of ZO can be used for the prevention and treatment of AAD.

## Figures and Tables

**Table 1:** Chemical properties of ZO extract.

**Chemical Properties**	**Mole (%)**
Neutral sugar	86.4 ± 5.1
Uronic acid	8.8 ± 0.8
Protein	4.0 ± 0.1
Polyphenol	0.8 ± 0.2
KDO-like material	not detected
**Component Sugar**	**Mole (%)**
Mannose	-
Rhamnose	-
Glucuronic acid	-
Galacturonic acid	-
Glucose	97.3 ± 0.6
Galactose	2.7 ± 0.9
Xylose	-
Arabinose	-
Fucose	-

-, Not detected.

**Table 2:** Scoring criteria for lincomycin-induced antibiotic-associated diarrhea in BALB/c mice.

Scores	Diarrhea Status
0	Normal (no diarrhea)
1	Loose, light colored and non-sticky perianal stool status; general mental state
2	Adhesion of stool at the anus; mental depression; no appetite for food; weight loss

**Table 3:** *Zingiber officinale* Roscoe freeze-dried yield.

Sample Name	Origin	Freeze-Dried Extract (g)	Yield (%)
*Zingiber officinale* Roscoe	Peru	10.3	34.3

**Table 4:** Analytical conditions for high-performance liquid chromatography (HPLC) for evaluating monosaccharide composition.

Pump	Liquid chromatography LC-20AD (Shimadzu Corporation, Kyoto, Japan)
Detector	UV/VIS detector (Shimadzu Corporation, Kyoto, Japan)
Column	Acclaim TM 120 C18 (Thermo Scientific, Sunnyvale, CA, USA)
Column size	4.6 × 250 mm
Column temp.	30 °C
Flow rate	1 mL/min
Eluent	0.1 M sodium phosphate buffer (pH 6.7): acetonitrile (82:18)
Injection vol.	10 μL
Integrator	Shimadzu data module (Shimadzu Corporation, Kyoto, Japan)

## Data Availability

Data are contained within the article.

## Supplementary Materials

The following supporting information can be downloaded at: https://www.mdpi.com/article/10.3390/molecules29030732/s1, Figure S1: HPLC chromatogram of ZO monosaccaharide composition.
