# Supplementary figures and images for: Ameliorative Effects of Zingiber officinale Rosc on Antibiotic-Associated Diarrhea and Improvement in Intestinal Function
Source: Molecules. 2024 Feb 5;29(3):732. doi: 10.3390/molecules29030732 (PMC10856109; doi:10.3390/molecules29030732)

## Standards material

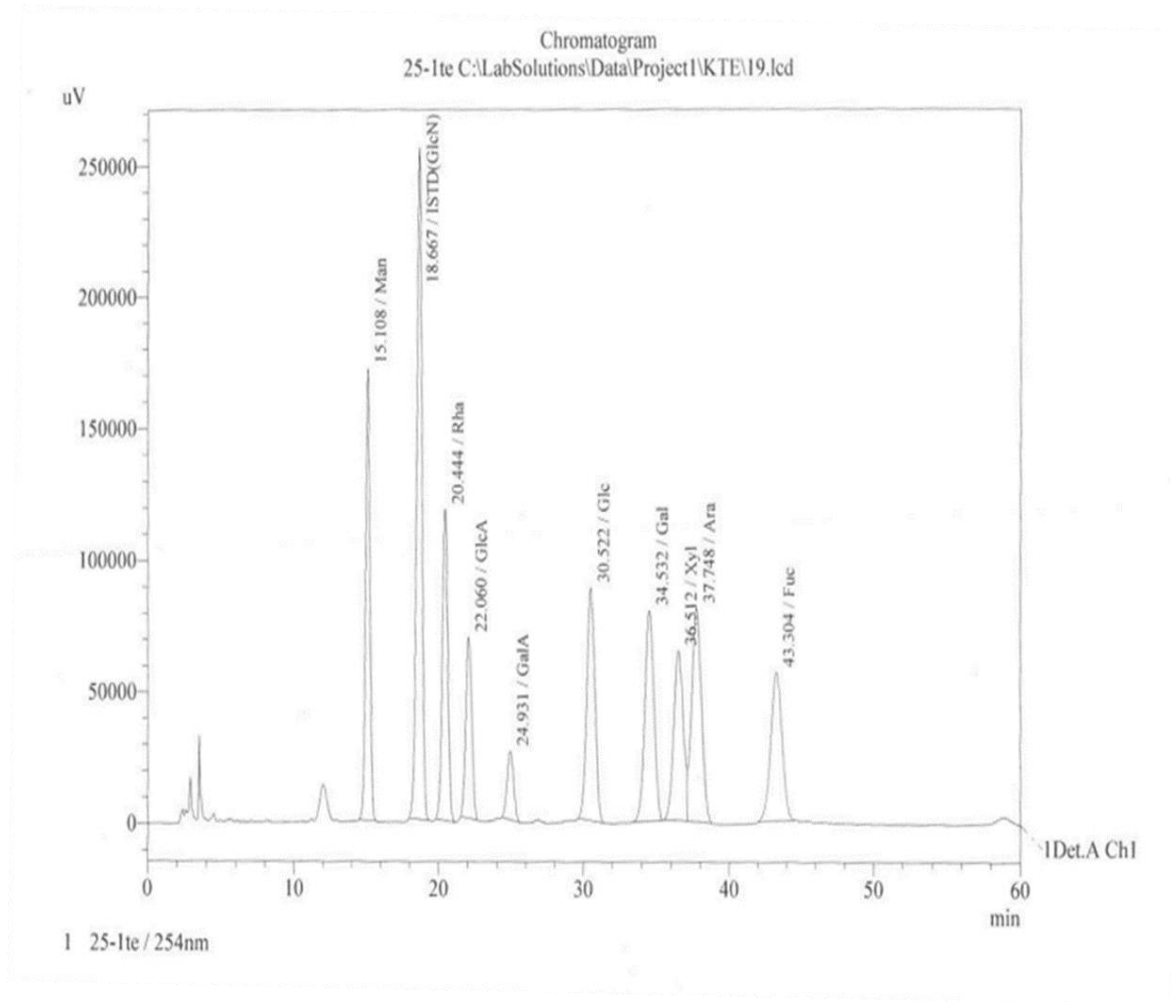

## ZO extract

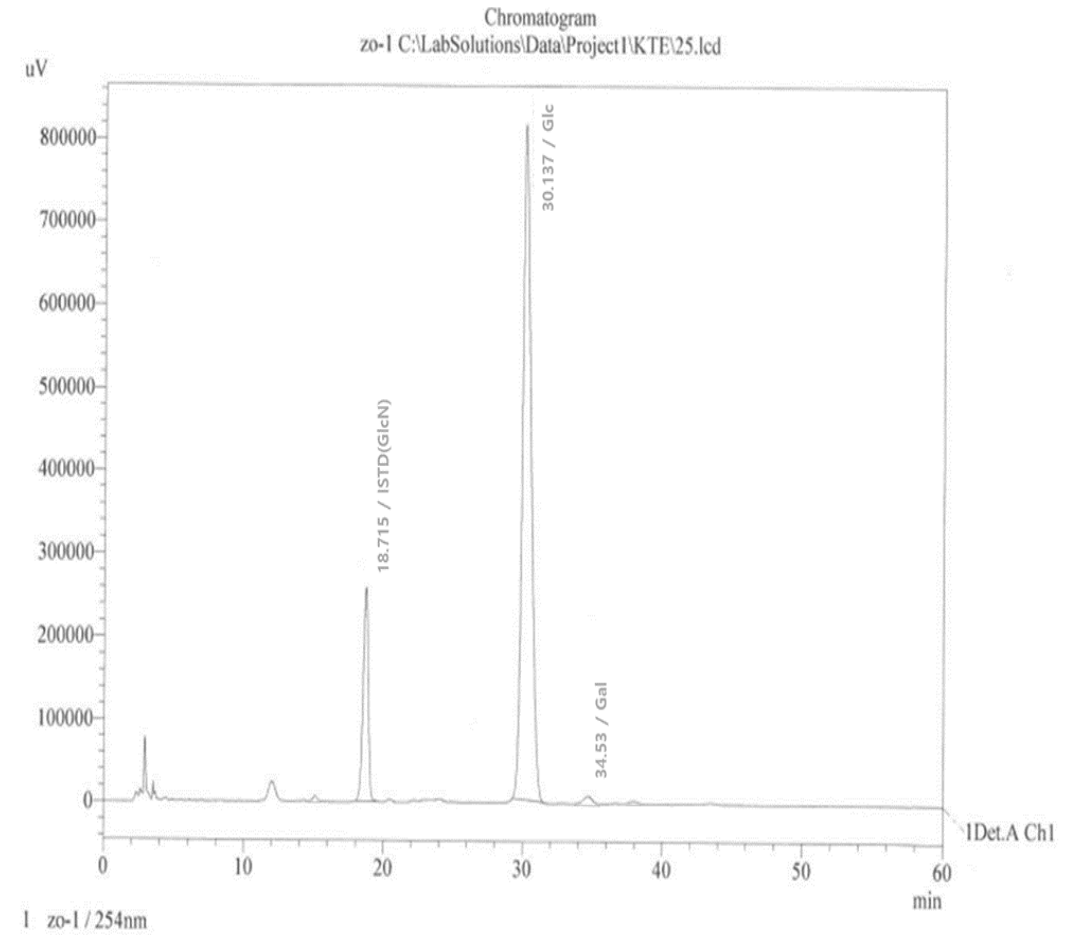

Supplement: Supplementary file 1 [file molecules-29-00732-s001.zip › molecules-2818511-supplementary.pdf]
